# Exploration of the heterogeneity and interaction of epithelial cells and NK/T-cells in Laryngeal Squamous Cell Carcinoma based on single-cell RNA sequencing data

**DOI:** 10.1016/j.bjorl.2023.02.003

**Published:** 2023-02-14

**Authors:** Yanan Liu, Zhiguang Gao, Cheng Peng, Xingli Jiang

**Affiliations:** Heilongjiang Provincial Hospital Affiliated to Harbin Institute of Technology, Department of Otorhinolaryngology, Harbin, Heilongjiang, PR China

**Keywords:** Laryngeal Squamous Cell Carcinoma, Heterogeneity, Epithelial cells, NK/T-cells

## Abstract

**Objectives:**

We aimed to explore the heterogeneity and differentiation trajectories of epithelial cells and NK/T-cells in Laryngeal Squamous Cell Carcinoma (LSCC).

**Methods:**

We downloaded the GSE150321 data set containing LSCC01 and LSCC02 samples single cell RNA data from Gene Expression Omnibus. The UMAP analysis was performed to identify the cell subpopulations and cell locations of subpopulations. Seurat package was used to analyze the differential expression of genes. The function of differential expression genes was analyzed using DAVID database. The monocle2 package was used to analyze differentiation trajectories. We used the CellChat package to observe the signaling pathways and ligand–receptor pairs for epithelial cells and NK/T-cells.

**Results:**

All the LSCC cells were divided into 16 subpopulation that included 7 epithelial cell subsets, 3 T-cell subsets. The function analysis indicated that epithelial cells and NK/T-cells mainly participated in different process, such as cell cycle, immune response, and cell migration. Then, the results of differentiation trajectory indicated that the ability of migration, and the activation of the immune system increases, while the ability of apoptosis, and glucose metabolic process decreases as pseudotime. Migration-related epithelial cells act on all T-cells via the CNTN2-CNTN2 ligand–receptor pair, which suggested that CNTN2 might be an important biomarker for regulating migration of epithelial cells.

**Conclusions:**

Our study characterized the heterogeneity of LSCC, which provided novel insights into LSCC and identified a new mechanism and target for clinical LSCC threapies.

**Evidence:**

IV.

## Highlights

• Characterized the heterogeneity of LSCC, which provided novel insights into LSCC.
• Igand-receptor interactions of epithelial and NK/T-cells to identify a new mechanism.
• CNTN2 might be a novel biomarker for LSCC.

## Introduction

Laryngeal Squamous Cell Carcinoma (LSCC) is a malignant tumor of the respiratory system with high morbidity and mortality.^1^ Approximately, 60% patients with LSCC were diagnosed at the advanced stage of tumor.^2^, ^3^ At present, the main therapies for LSCC are operation, chemotherapy and radiation.^4^, ^5^ Although treatment techniques have greatly improved over the past decade, they are still far from satisfactory. Tumor heterogeneity may be an important cause of ineffective treatment.^6^, ^7^ Understanding the composition and molecular characteristics of LSCCs at the cellular level may help address this issue.

Due to cellular heterogeneity, the genetic information of cells of the same phenotype may differ significantly, and much low-abundance information is lost in the overall characterization. Single-cell sequencing technology is explored to make up for the limitations of traditional high-throughput sequencing. LSCC is characterized by complex relations between stromal, epithelial, and immune cells within the Tumor Microenvironment (TME).^8^ Previous study has reported that the “epithelial-immune” dual-characterized tumor cell subsets have stronger immunosuppressive and regulatory functions for T-cells in the immune microenvironment. Flow cytometry analysis confirmed that the proportion of infiltrating T-cells expressing co-inhibitory molecule receptors in the immune microenvironment of nasopharyngeal carcinoma was positively correlated with tumor cells with dual characteristics of “epithelial-immunity”. Further analysis indicated that dual-characterized tumor cells have the ability to inhibit the secretion of IFN-γ by CD8+ TILs, which may be an important reason for the exhaustion of tumor-infiltrating T-cells.^9^, ^10^

Thus, our study mainly explored the heterogeneity and differentiation trajectories of epithelial cells and NK/T-cells in LSCC tissues. For epithelial cells, we found that as the differentiation trajectory progressed, epithelial cells were mainly divided into two categories, one mainly involved in cell proliferation and the other mainly involved in immune activation responses. As for NK/T-cells, we found that with the progression of the differentiation trajectory, T-cells are also mainly divided into two categories, one tends to immune activation and killing tumor cells, and the other tends to be exhausted. Furthermore, we divided epithelial cells into proliferative epithelial cells, immune-related epithelial cells, and migration-related epithelial cells according to their functions. For NK/T-cells, we divided them into cytotoxic T-cells and exhausted T-cells. Finally, we explored the ligand–receptor interaction of different types of epithelial cells and NK/T-cells, thereby revealing the molecular mechanism of mutual activation and inhibition between epithelial cells and NK/T-cells in LSCC tissue.

## Methods

### Data source

The GSE150321 dataset was downloaded from gene expression omnibus (GEO, https://www.ncbi.nlm.nih.gov/geo/) including LSCC01 and LSCC02 tumor samples obtained from 66-year-old patient and a 64-year-old male patient. Neither patient received clinical treatment.

### Pre-processing of the data

The Seurat package^11^ (version 4.1.0) in R language (version 4.1.2) was performed to read the single cell gene expression matrix, based on which we conducted the following analysis. First, we delete the cells with <200 expressed genes and genes with <3 cells present. The PercentageFeatureSet function was performed to caLSCCulate the propertion of mitochondrial genes in cells. Next, the cells meet the threshold of UMI ranged from 200 to 6000, mitochondrial gene ratio <50% were included in this study. Then, the data was normalized using SCTransform function, analyzed for principal component analysis using RunPCA function, and removed batch effects using harmony package.^12^ After that, according to the top 30 principal components, the FindNeighbors function was carried out to construct the public nearest neighbor graph based on euclidean distance metric. The clustering cells were visualized using RunUMAP function, which was divided using FindClusters function. The resolution set to 0.26 for all the cells and epithelial cells, and 0.1 for T-cell. Finally, we performed DoubletFinder package^13^ to remove double cells. Subsequently, a total of 711 double cells were removed and the rest 11,990 cells were included in our study for further analysis.

### Identification of differentially expressed genes in cell subsets

To identify the Differentially Expressed Genes (DEGs) in cell subsets, the FindAllMarker function in Seurat package was performed to caLSCCulate the significance of the DEGs with the parameter of min.pct = 0.25, logfc.threshold = 0.5, and only.pos = TRUE. Then, to further understand the function of DEGs, we searched the biological process of DEGs from DAVID database^14^ (https://david.ncifcrf.gov/) using the threshold of *p* < 0.05.

### Differentiation trajectory analysis of single cells

The monocle2 in R package^15^ (version 2.22.0) was used to performed the pseudo-time trajectory analysis of single cells. The data processed using Seurat was regarded as the gene-cell matrix of raw UMI counts. Then, we used the newCellDaraset function to produce an object for pseudo-time trajectory analysis (expressionFamily = negbinomial.size). Subsequently, we included genes with the mean expression value was above 0.1 and their expression detected in at least 10 cells for pseudo-time trajectory analysis. Then, the differential GeneTest function was used to screen the DEGs, and the genes with q-value <1e-30 was used to dimensionality reduction. Next, the reduce Dimension function was used for dimensionality reduction with the parameter of reduction_method = “DDRTree” and max_components = 2. We used the orderCells function to construct pseudotime trajectories and arrange cells, and plot_cell_trajectory to sort and visualize cells. After that, differential GeneTest with the parameter of fullModelFormulaStr = “∼sm.ns(Pseudotime)” was performed to caLSCCulate the genes changed with pseudotime. The genes were visualized using “plot_pseudotime_heatmap”, which was grouped based on expression pattern. For branch analysis of single-cell differentiation trajectories, we used the BEAM function to count genes with significantly different expression patterns between the two branches. Also, the genes were visualized using “plot_genes_branched_heatmap”, which were grouped according to expression patterns.

### Analysis of cell–cell communication

CelLSCChat^16^ is an analytical tool for exploring ligand–receptor interactions of specific signaling pathways between cells. To explore the ligand–receptor interactions between different types of epithelial cells and T-cells, the CelLSCChat in R package (Version 1.1.3) was used to perform the analysis of cell–cell communication.

### Statistical analysis

All analysis procedures and image generation were performed by R language (version 4.1.2), and *p* < 0.05 was considered to be statistically significant.

## Results

### Cellular composition in LSCC tissue

**Figure 1:**
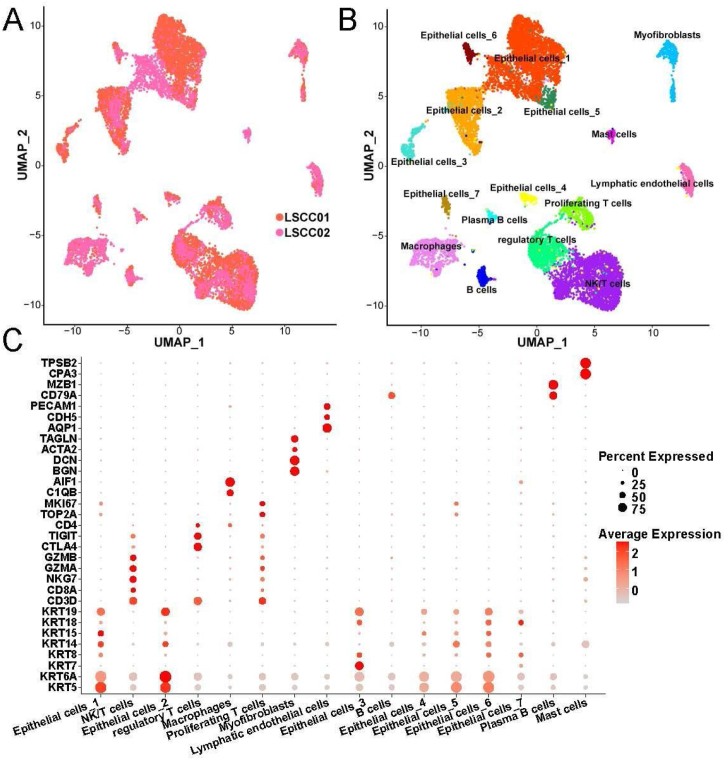
Grouping and identification of cells in laryngeal cancer tissues. (A) Distribution of cells from different samples. (B) Location distribution of UMAP in different types of cells. (C) Bubble plot of marker genes in different cell types.

Through standardization by SCTransform, UMAP dimensionality reduction and clustering and removal of batch effects by harmony, we found that the cells in LSCC001 and LSCC002 were evenly distributed together (Fig. 1A). Next, all the cells were divided into 16 subpopulation that included 7 epithelial cell subsets, 3 T-cell subsets (Fig. 1B). Besides, the 7 epithelial cell subsets were found related to their high expression of epithelial cell markers, such as KRT5, KRT14 and KRT19. T-cell subsets have high expression of the marker gene CD3D. Meanwhile, regulatory T-cells were correlated to high expression of CTLA4, TIGIT and other genes that inhibit the activity of immune cells, proliferating T-cells highly expressed TOP2A, MKI67 and other cell proliferation-related genes. These results indicated that the T-cell subset was in an active state of cell division. We have listed the high expression genes in each cell subset as Table S1.

### Heterogeneity and differentiation trajectories of epithelial cells in LSCC

**Figure 2:**
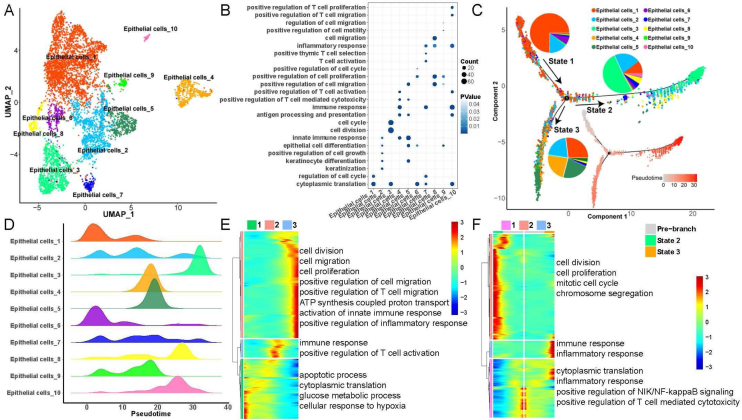
Heterogeneity and differentiation trajectories of epithelial cells in laryngeal carcinoma tissue. (A) UMAP plot of epithelial cells divided into 10 subpopulations. (B) Bubble plot of the enrichment of highly expressed genes in 10 epithelial cell subpopulations to representative biological processes. (C) Differentiation trajectories of 10 epithelial cell subsets and the proportion of each type of epithelial cell in each state. (D) Mountain plot of density distribution of 10 epithelial cell subsets in pseudotime. (E) Variation with pseudotime the biological process to which the genes are mainly enriched. (F) The biological process to which the branch-related genes between State 2 and State 3 are mainly enriched.

To explore heterogeneity and differentiation trajectories of epithelial cells in LSCC, we merged all the epithelial cells and re-clustered them into a total of 10 cell subsets (Fig. 2). We listed the genes with high expression in epithelial cells as Table S2. Then, we studied the biological process of the highly expressed genes in each epithelial cell. Interestingly, we found that epithelial cell subsets 1, 3, 6 were mainly involved in cell cycle and cell division related functions. Epithelial cell subsets 2, 4, 5, 7 and 10 were mainly involved in immune-related functions such as immune response, T-cell activation, and inflammatory response. Epithelial cell subsets 8 and 9 were mainly involved in cell migration-related functions (Fig. 2B).

Moreover, we further explored the differentiation trajectory of the epithelial cells, the results indicated that the subgroup 3 of epithelial cells in the branch of State 2 accounted for the largest proportion, while the subgroups 1, 2, 4 and 5 of epithelial cells in the branch of State 3 accounted for the largest proportion. This result implies that epithelial cells are mainly differentiated into two types in the process of differentiation and evolution, one is mainly involved in cell proliferation, and the other is mainly involved in immune activation (Fig. 2C‒D). Besides, with the progress of epithelial cell differentiation, the ability of cell division and migration, and the activation of the immune system gradually increased, while the ability of apoptosis, cytoplasmic translation, and glucose metabolic process gradually decreased (Fig. 2E). Finally, we explored the differences in gene expression between the two branches of State 2 and State 3, the results indicated that with the pseudotime, the expression of genes related to cell division and mitotic cell cycle in State 2 gradually increased, while the expression of genes associated with immune response and inflammatory response in State 3 also gradually increased (Fig. 2F).

### Heterogeneity and differentiation trajectories of NK/T-cells in LSCC

**Figure 3:**
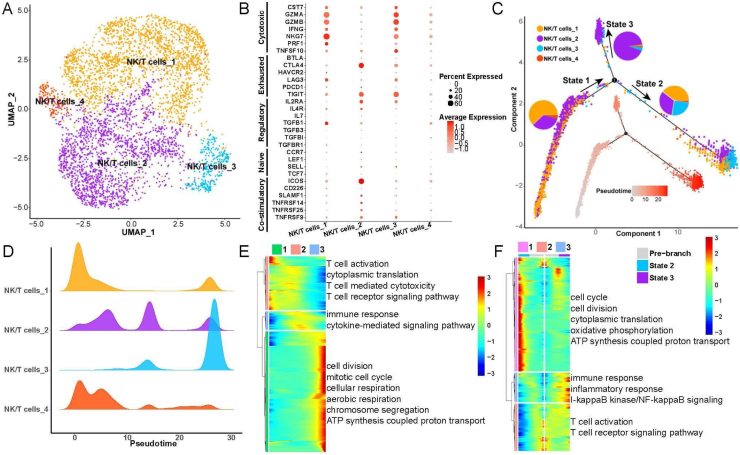
Heterogeneity and differentiation trajectories of T-cells in laryngeal cancer tissues. (A) UMAP plot of T-cells divided into 4 subpopulations. (B) Bubble plot of known different T-cell marker genes in 4 subpopulations. (C) Differentiation trajectories of the 4 T-cell subsets and the proportion of each T-cell in each state. (D) Mountain plot of the density distribution of the 4 T-cell subsets in pseudotime. (E) Genes as a function of pseudotime the biological process that is mainly enriched. (F) The biological process that the branch-related genes between State 2 and State 3 are mainly enriched.

To explore heterogeneity and differentiation trajectories of NK/T-cells in LSCC, we merged all the epithelial cells and re-clustered them into 4 cell subsets (Fig. 3A). We found that the NK/T-cell subsets 1 and 3 had high expression of GZMA, GZMB, NKG7 and other cytotoxic T-cell marker genes, while NK/T-cell subset 2 were highly expressed CTLA4, TIGIT and other exhausted T-cell marker genes (Fig. 3B). We have listed the gene markers of NK/T-cell at Table S3.

Next, we explored the differentiation trajectories of NK/T-cells, the results indicated that NK/T-cell subsets 1, 2, 3 in State 2 had the largest proportion, and the NK/T-cell subset 2 in State 3 had the biggest proportion (Fig. 3C and D), indicating that during the differentiation and evolution of NK/T-cells, one type tends to activate immune cells and kill tumor cells, and the other type tends to be exhausted. Then we found that with the progress of NK/T-cell differentiation, the ability of cell division and aerobic respiration gradually increased, and the ability of T-cell activation gradually decreased (Fig. 3E). Furthermore, we analyzed the differential expression of genes between State 2 and State 3, the results found that with the change of pseudotime, the expression of genes related to cell cycle, oxidative phosphorylation in State 2 were gradually increased, while the expression of genes related to immune response, inflammatory response, T-cell activation were also improved (Fig. 3F).

### Communication analysis of epithelial cells and T-cells in LSCC

Finally, we explored the ligand–receptor interactions of different types of epithelial cells and NK/T-cells in LSCC tissues. In particular, we pooled epithelial cell subsets 1, 3, 6 together and named proliferative epithelial cells; epithelial cell subsets 2, 4, 5, 7, 10 pooled together and named immune-related epithelial cells; epithelial cells Cell subsets 8, 9 were merged together and named migration-related epithelial cells. For NK/T-cells, we pooled NK/T cell subsets 1 and 3 together and named cytotoxic T-cells; NK/T-cell subset 2 named exhausted T-cells.

**Figure 4:**
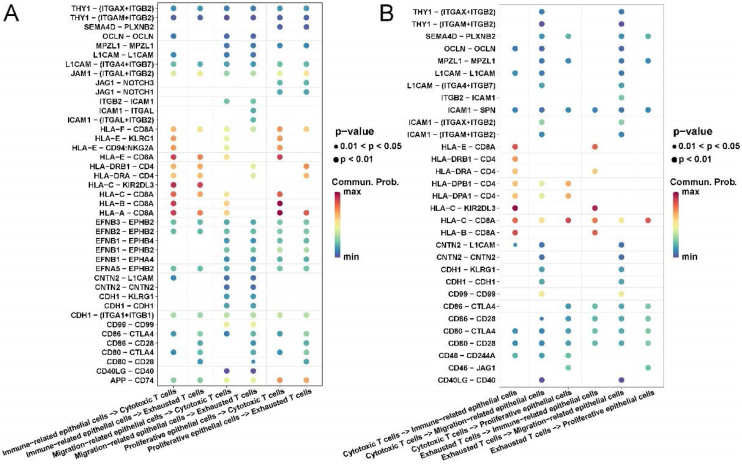
Communication analysis of cell contact types between epithelial cells and immune cells in laryngeal cancer tissues. (A) Ligand–receptor bubble diagram of different types of epithelial cells acting on T-cells. (B) Different types of T-cells acting on epithelial cells ligand–receptor bubble diagram.

Interestingly, we found that immune related and proliferative epithelial cells activate cytotoxic T-cells by acting on CD8A through HLA-A, B, C. Migration-related epithelial cells act on all T cells through ligand–receptor pairs such as CNTN2-CNTN2, CDH1-KLRG1, CDH1-CDH1. All epithelial cells had stronger CD86-CTLA4 and CD80-CTLA4 interactions with exhausted T-cells than with cytotoxic T-cells (Fig. 4A).

Conversely, all T-cells act on immune-related epithelial cells through ligand–receptor pairs such as HLA-C-KIR2DL3, HLA-B-CD8A, and HLA-E-CD8A. All T-cells act on migration-related epithelial cells through ligand–receptor pairs such as CNTN2-L1CAM, CNTN2-CNTN2, CDH1-KLRG1, CDH1-CDH1, CD99-CD99. Exhausted T-cells act on all epithelial cells through ligand–receptor pairs such as CD86-CTLA4, CD86-CD28, CD80-CTLA4, CD80-CD28 (Fig. 4B).

## Discussion

Among all the types of head neck squamous cell carcinoma, LSCC has the highest rate of morbidity and mortality.^17^ Although therapies for LSCC have improved in recent decades, the clinical outcomes are unsatisfied. Cellular heterogeneity is considered an important factor for the limited ineffective treatmen.^18^ Ligand–receptor complex-mediated cell–cell communication is critical for coordinating multiple biological processes including development, differentiation, and inflammation.^19^ Thus, our study analyzed the cell–cell communication of epithelial cells and T-cells to explore the mechanism of LSCC.

In our study, we found that the epithelial cells were divided into 10 cell subtypes, which were related different function such as cell cycle, cell division, immune response, T-cell activation, and cell migration. We further analyzed the differentiation trajectory. The results indicated that the ability of cell division and migration and the activation of the immune system gradually increases, while the ability of apoptosis, cytoplasmic translation, and glucose metabolic process gradually decreases as the differentiation of epithelial cells progresses. These results were consistent with the real differentiation trajectory of LSCC.^20^, ^21^ NK/T-cells play an important role in improving clinical outcomes and regulating response to chemotherapy in kinds of cancers, such as liver cancer,^22^ ovarian cancer,^23^ and breast cancer.^24^ We also analyzed the function of NK/T cells in LSCC, the results indicated that NK/T-cells were involved in the function of immune response, inflammatory response, T-cell activation. Besides, as the differentiation of NK/T-cells, their different functions were changed that consistent with the development of LSCC.^25^ All the results suggested that epithelial cells and NK/T-cells had complex heterogeneity, which were the main cause of unsatisfactory outcome in clinically. Meanwhile, previous report also indicated that the heterogeneity of epithelial-derived cells and immune cells is the main reason for the heterogeneity of laryngeal carcinoma.^26^, ^27^

To explore the mechanism of LSCC, we studied the ligand–receptor interactions of different types of epithelial cells and NK/T-cells in LSCC. All the epithelial cells were divided into proliferative, immune-related and migration-related epithelial cells. The association between the expression levels of HLAs and the clinical course of many malignancies reflects their critical role in the recognition and elimination of malignant cells by cognate T-cells and NK cells.^28^ We found that immune related and proliferative epithelial cells activate cytotoxic T-cells by acting on CD8A through HLA-A, B, C. Previous studies also found that similar results that decreased HLA-A, -B, and -C expression were found in stage I‒II tumors via regulating the expression of CD8A.^29^ Interestingly, migration-related epithelial cells act on all T-cells via the CNTN2-CNTN2 ligand–receptor pair, and also, all T-cells act on migration-related epithelial cells via the CNTN2-CNTN2 ligand–receptor pair. We inferred that CNTN2 might be an important biomarker for regulating migration of epithelial cells. CNTN2 encodes a member of the contactin family of proteins, part of the immunoglobulin superfamily of cell adhesion molecules. A mutation in CNTN2 may be associated with adult myoclonic epilepsy. Recently, CNTN2 was reported to involve in the development of some cancers, such as hepatocellular carcinoma,^30^ glioma,^31^ and lung adenocarcinoma. However, few studies have reported the role of CNTN2 in the procession of LSCC. Our study suggested CNTN2 might regulate the migration of LSCC epithelial cells via mediating T-cells. This result provided a novel direction for seeking effective therapies for LSCC.

## Conclusions

Our study characterized the heterogeneity of LSCC, which provided novel insights into LSCC. Futhermore, we analyzed the ligand–receptor interactions of epithelial cells and NK/T-cells to identify a new mechanism and target for clinical LSCC therapies.

## Authors’ contributions

Yanan Liu and Xingli Jiang contributed to the research conception and design. Zhiguang Gao and Yanan Liu performed the data collection. Statistical analysis was performed by Cheng Peng. Yanan Liu drafted the manuscript. Xingli Jiang revised the manuscript.

## Funding

This work was supported by Scientific Research Project of Heilongjiang Health and Family Planning Commission (2019‒147; 2018‒021).

## Statement of ethics

Not applicable.

## Data availability

All data generated or analyzed during this study are included in this article and its Supplementary material files. Further enquiries can be directed to the corresponding author.

## Conflicts of interest

The authors declare no conflicts of interest.
